# Genetic diversity and population structure analysis of *Saccharum* and *Erianthus* genera using microsatellite (SSR) markers

**DOI:** 10.1038/s41598-018-36630-7

**Published:** 2019-01-23

**Authors:** Ahmad Ali, Yong-Bao Pan, Qin-Nan Wang, Jin-Da Wang, Jun-Lü Chen, San-Ji Gao

**Affiliations:** 1 National Engineering Research Center for Sugarcane, Fujian Agriculture and Forestry University, Fuzhou, Fujian 350002 China; 2 USDA-ARS, Sugarcane Research Unit, Houma, LA 70360 USA; 3 Guangdong Bioengineering Institute (Guangzhou Sugarcane Industry Research Institute), Guangzhou, Guangdong 510316 China

## Abstract

In order to understand the genetic diversity and structure within and between the genera of *Saccharum* and *Erianthus*, 79 accessions from five species (*S. officinarum*, *S. spontaneum*, *S. robustum*, *S. barberi*, *S. sinense*), six accessions of *E. arundinaceus*, and 30 *Saccharum* spp. hybrids were analyzed using 21 pairs of fluorescence-labeled highly poloymorphic SSR primers and a capillary electrophoresis (CE) detection system. A total of 167 polymorphic SSR alleles were identified by CE with a mean value of polymorphic information content (PIC) of 0.92. Genetic diversity parameters among these 115 accessions revealed that *Saccharum* spp. hybrids were more diverse than those of *Saccharum* and *Erianthus* species. Based on the SSR data, the 115 accessions were classified into seven main phylogenetic groups, which corresponded to the *Saccharum* and *Erianthus* genera through phylogenetic analysis and principle component analysis (PCA). We propose that seven core SSR primer pairs, namely, SMC31CUQ, SMC336BS, SMC597CS, SMC703BS, SMC24DUQ, mSSCIR3, and mSSCIR43, may have a wide appicability in genotype identification of *Saccharum* species and *Saccharum* spp. hybrids. Thus, the information from this study contibites to manage sugarcane genetic resources.

## Introduction

Sugarcane (*Saccharum* spp.) plays a vital role as a primary sugar-producing crop (sugar 80%) and has major potential as a renewable bioenergy crop (ethanol 50%) in world agriculture^1^. The *Saccharum* complex contains six main species: the two wild species are *S. spontaneum* and *S. robustum*, and the four cultivated species are *S. officinarum*, *S. sinense*, *S. barberi* and *S. edule*^2^. In addition, *Erianthus arundinaceus* is a species of *Erianthus* genus with strong abiotic stress tolerance and could be widely used for modern sugarcane breeding and a potential bioenergy plant^3^. Currently, sugarcane commercial breeding populations in the world share a narrow genetic base due to their common origins from a number of popular cultivars, such as POJ2878, Co419 and NCo310 which were achieved in the early 1900s^2^. Furthermore, these exotic varieties were developed from complex interspecific hybridization through Noblization Breeding process among wild clones of *S. spontaneum* and *S. officinarum*^4^. There is still a great attention among sugarcane breeders in broadening the genetic base of the crop and also in taping into the gene pool of the wild relatives to enhance stress-resistance and sucrose content^5^.

Since the late 1980s, sugarcane breeders and geneticists have discovered and use several DNA molecular markers including amplified fragment length polymorphisms (AFLP), restriction fragment length polymorphisms (RFLP), random amplification of polymorphic DNAs (RAPD), single nucleotide polymorphism (SNP), simple sequence repeats (SSRs), inter simple sequence repeat (ISSRs), and expressed sequence tag- simple sequence repeat (EST-SSRs) to improve *Saccharum* breeding^6^. Among these molecular markers, SSR (microsatellite) markers have been widely used to study sugarcane genetic diversity^7^, genetic mapping^8^, cross-transferability^9^, paternity analysis^10^, segregation analysis^11^, and marker-assisted selection^12^. SSR primer pairs are considered the most capable marker for plant genetics and breeding programs, because of co-dominant, multi-allelic nature, and relatively abundant with an excellent genome coverage^13^.

Early molecular marker research focused on the origin of wild *Saccharum* species. Lu *et al*.^14^ proposed a hybrid origin for *S. barberi* and *S. sinense* from natural hybridization between *S. spontaneum* and *S. officinarum*, based on a factorial correspondence analysis of RFLP markers. Subsequently, these results were supported by Irvine^15^ and Selvi *et al*.^16^ using SSR markers and by D’Hont *et al*.^17^ utilizing genomic *in situ* hybridization (GISH). Based on analysis of agronomic traits and mitochondrial profiles, S*. barberi* and *S. sinense* were placed in adjacent clusters, but apart from *S. robustum*^18–20^. Later on a number of reports focused on the analysis of genetic diversity and population structure among commercial *Saccharum* spp. hybrids varieties^7,21–25^ and among *S. spontaneum* populations with different ploidy levels in China^26^. Therefore, there has been an increasing interest among sugarcane breeders to investigate the genetic diversity of parental resources and to broaden the genetic base by tapping into the gene pools of the wild relatives^27–29^.

To better understand the genetic background of these euploid sugarcane clones, this study aimed to characterize the genetic diversity and population structure of 115 accessions belonging to *S. officinarum*, *S. spontaneum*, *S. robustum*, *S. barberi*, *S. sinense*, *E. arundinaceus*, and *Saccharum* spp. hybrids. The results may provide invaluable information for the better utilization of *Saccharum* and *Erianthus* wild germplasms at different ploidy levels in sugarcane breeding.

## Results

### Total alleles amplification of 21 SSR markers

A total of 167 SSR alleles were amplified from the DNA of 115 accessions including five *Saccharum* species, *E. arundinaceus*, and 30 clones of *Saccharum* spp. hybrids with the 21 fluorescence-labeled SSR primer pairs and capillary electrophoresis (CE) detection system. We could not find in our CE data the 16 SSR alleles reported earlier by Pan^30^, but instead, we have found 38 new SSR alleles that were never reported before (Table 1). Furthermore, the numbers of new and absent SSR alleles detected in this study were greater than the 20 new and 13 absent SSR alleles reported previously by Ali *et al*.^7^.

**Table 1:** The general utility and amplification profile of 21 SSR primer pairs based on a capillary electrophoresis (CE) detection platform.

No.	Markers^a^	Size range (bp)	Number of original bands	Number of detected bands	Absent alleles (bp)^b^	New alleles (bp)^b^	PIC
1	SMC119CG	104–135	5	6	ND	104	0.92
2	SMC1604SA	105–130	6	7	109,124	105,107,110	0.93
3	SMC1751CL	132–160	5	7	ND	132,138	0.94
4	SMC18SA	135–150	5	6	ND	135	0.93
5	SMC22DUQ	125–165	7	8	125	142146	0.94
6	SMC24DUQ*	124–150	6	10	ND	124,133,139,150	0.95
7	SMC278CS	138–182	9	9	140,153,176	138,164,172	0.94
8	SMC31CUQ*	135–180	11	12	138	135,169	0.95
9	SMC334BS	143–165	6	7	ND	143	0.94
10	SMC336BS*	140–185	11	10	154	0	0.95
11	SMC36BUQ	100–125	3	4	ND	102	0.80
12	SMC486CG	220–245	5	5	227	235	0.91
13	SMC569CS	165–225	5	5	167,170,222	165,202	0.82
14	SMC597CS*	140–180	11	13	ND	150,152	0.95
15	SMC703BS*	200–225	9	11	ND	204,218	0.95
16	SMC7CUQ	140–170	6	7	158	143,160	0.90
17	SMC851MS	125–145	6	7	ND	138	0.94
18	mSSCIR3*	140–190	10	10	141,145	149,169	0.95
19	mSSCIR43*	200–255	9	10	209	203,229	0.95
20	mSSCIR66	120–145	4	7	ND	125,136,142	0.94
21	mSSCIR74	210–232	5	6	ND	214	0.94
	Total		144	167			
	Average			7.95			0.92

^a^Core primer was marked with asterisk (*).

^b^Absent and new alleles detected in this study comparing with the 144 alleles Pan^30^; ND, no data.

The number of alleles detected by the CE system varied from as few as four (SMC36BUQ) to as many as 13 (SMC597CS), with an average of 7.95 per SSR primer pair. Seven SSR primer pairs, namely SMC24DUQ, SMC31CUQ, SMC336BS, SMC597CS, SMC703BS, mSSCIR3 and mSSCIR43, were highly polymorphic, each producing 10 to 13 alleles. Other eleven SSR primer pairs, namely, SMC119CG, SMC1604SA, SMC1751CL, SMC18SA, SMC22DU, SMC278CS, SMC334BS, SMC7CUQ, SMC851MS, mSSCIR66 and mSSCIR74, were moderately polymorphic, each producing six to nine alleles. The remaining three SSR primer pairs, namely, SMC36BUQ, SMC486CG and SMC569CS, were less polymorphic by producing less than six alleles each (Table 1). The PIC values of these primer pairs ranged from 0.80 (SMC36BUQ) to 0.95 (SMC24DUQ, SMC31CUQ, SMC336BUQ, SMC597CS, SMC703BS, mSSCIR3, mSSCIR43) with an average of 0.92 (Table 1).

The PIC values of each *Saccharum* and *E. arundinaceus* species were also calculated in our study. The maximum PIC value was 0.95 for mSSCIR3 on *S. spontaneum* and the minmum PIC value was 0.28 for SMC119CG on *E. arundinaceus*. Generally, higher PIC values were found in *Saccharum* spp. hybrids with an average value of 0.87, followed by an average PIC value of 0.86 in *S. spontaneum* (Table 2).

**Table 2:** Polymorphism information content (PIC) of 21 SSR primer pairs analysed using 115 accessions from *Saccharum*, *Erianthus*, and *Saccharum* spp. hybrids.

No.	SSR markers	*S. spontaneum*	*S. officinarum*	*S. barberi*	*S. robustum*	*S. sinense*	*Saccharum* spp. hybrids	*E. arundinaceus*
1	SMC119CG	0.82	0.91	0.79	0.73	0.76	0.86	0.28
2	SMC7CUQ	0.88	0.84	0.67	0.85	0.62	0.84	0.38
3	SMC18SA	0.86	0.82	0.80	0.81	0.70	0.91	0.50
4	SMC22DUQ	0.88	0.89	0.83	0.73	0.61	0.88	0.59
5	SMC24DUQ	0.85	0.88	0.83	0.73	0.67	0.88	0.55
6	SMC31CUQ	0.90	0.88	0.64	0.83	0.81	0.91	0.76
7	SMC36BUQ	0.79	0.68	0.59	0.61	0.63	0.82	0.72
8	SMC278CS	0.93	0.77	0.65	0.81	0.81	0.86	0.78
9	SMC334BS	0.91	0.72	0.84	0.71	0.67	0.84	0.38
10	SMC336BS	0.92	0.88	0.84	0.71	0.71	0.88	0.73
11	SMC486CG	0.77	0.72	0.80	0.68	0.72	0.86	0.45
12	SMC569CS	0.61	0.77	0.49	0.73	0.61	0.83	0.55
13	SMC597CS	0.83	0.86	0.82	0.72	0.35	0.93	0.78
14	SMC703BS	0.82	0.60	0.56	0.77	0.64	0.92	0.88
15	SMC851MS	0.89	0.91	0.83	0.72	0.83	0.88	0.79
16	SMC1604SA	0.89	0.83	0.66	0.82	0.62	0.86	0.74
17	SMC1751CL	0.88	0.84	0.79	0.80	0.72	0.82	0.78
18	mSSCIR3	0.95	0.91	0.78	0.86	0.85	0.89	0.89
19	mSSCIR43	0.92	0.90	0.82	0.85	0.58	0.91	0.88
20	mSSCIR66	0.86	0.86	0.82	0.82	0.80	0.82	0.76
21	mSSCIR74	0.92	0.92	0.81	0.81	0.78	0.83	0.79
	Average	0.86	0.83	0.75	0.77	0.69	0.87	0.66

### Genetic variability

**Figure 1:**
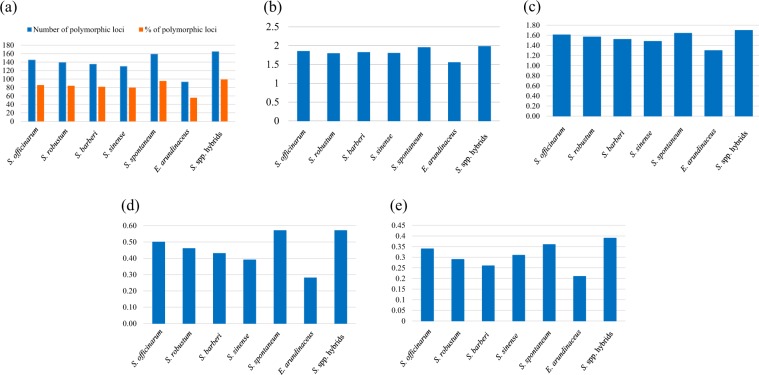
Statistical analysis of genetic variability among *Saccharum*, *Erianthus* and *Saccharum* spp. hybrids populations based on SSR data. Polymorphism index (*PI*) (**a**), Number of observed alleles (*Na*) (**b**), Number of effective alleles (*Ne*) (**c**), Shannon’s index (*I*) (**d**), and Nei’s genetic diversity (*h*) (**e**).

Using the CE detection system, an average of 138 polymorphic SSR bands was observed in each *Saccharum* or *E. arundinaceus* species. Among the five species of *Saccharum*, one species of *Erianthus*, and *Saccharum* spp. hybrids, both the highest number of polymorphic loci (NPL) and the highest percentage of polymorphic loci (PPL) were observed in *Saccharum* spp. hybrids population (*NPL* = 165, *PPL* = 98.8%), followed by *S. spontaneum* (*NPL* = 159, *PPL* = 95.21%), while the lowest number and percentage of polymorphic loci were found in *E. arundinaceus* (*NPL* = 93, *PPL* = 55.69%) (Fig. 1a). The highest number of observed alleles (*Na* = 1.98) was found in *Saccharum* spp. hybrids, while the lowest number of observed alleles (*Na* = 1.55) was found in *E. arundinaceus* (Fig. 1b). Morever, the highest number of effective alleles (*Ne* = 1.70) was found in the *Saccharum* spp. hybrids, followed by *S. spontaneum* (*Ne* = 1.64). The lowest number of effective alleles (*Ne* = 1.30) was observed in *E. arundinaceus* (Fig. 1c). Shannon’s index information of different populations ranged from 0. 28 (*E. arundinaceus*) to 0.57 (*Saccharum*. spp. hybrids). Analysis of Shannon’s index (*I*) showed that *Saccharum* spp. hybrids and *S. spontaneum* were different from the rest of other *Sccharum* species by sharing the highest shannon’s index value of 0.57. The lowest shannon’s diversity index value of 0.28 was observed in *S. sinense* (Fig. 1d). The Nei’s gene diversity (*h*) of the seven populations ranged from 0.21 to 0.39. The higher genetic diversity values of 0.39, 0.36 and 0.34 were observed in *Saccharum* spp. hybrids, *S. spontaneum* and *S. officinarum* populations, respectivaly; while the *E. arundinaceus* and *S. barberi* populations had the lower genetic diversity values of 0.21 and 0.26 (Fig. 1e).

### Principal Component Analysis (PCA)

**Figure 2:**
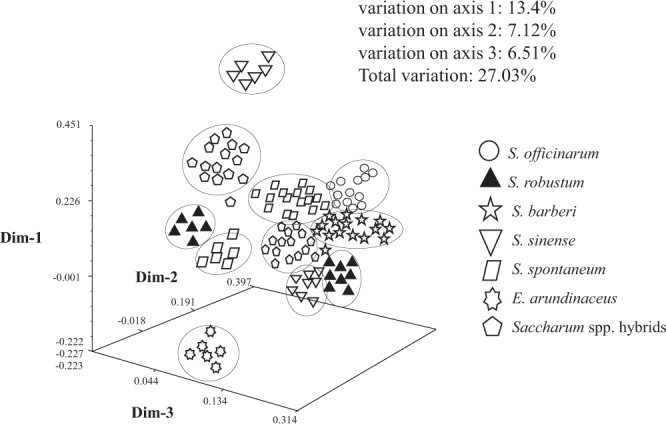
Three-dimensional principal component analysis (PCA) plot of *Saccharum*, *Erianthus*, and *Saccharum* spp. hybrids based on SSR data.

Principal component analysis (PCA) data for all 115 accessions are shown in Fig. 2. The analysis classified these accessions into evelen groups involving different *Saccharum* and *Erianthus* species to some extent, i.e., Group I-A and I-B (*Saccharum* spp. hybrids), Group II-A and II-B (*S. spontaneum*), Group III (*S. barberi*), Group IV-A and IV-B (*S. robustum*), Group V-A and V-B (*S. sinense*), Group VI (*S. officinarum*), and Group VII (*E. arundinaues*) (Fig. 2). The amount of variance accounted for by the globle three-dimensional plot is 13.4% of Dim1, 7.12% of Dim2, and 6.51% of Dim3, with a total of 27.03% for three dimensions. This is an acceptale fit, given the small amount of variability from the large number of accessions and SSR alleles used in the analysis.

### Phyolgenetic analysis

**Figure 3:**
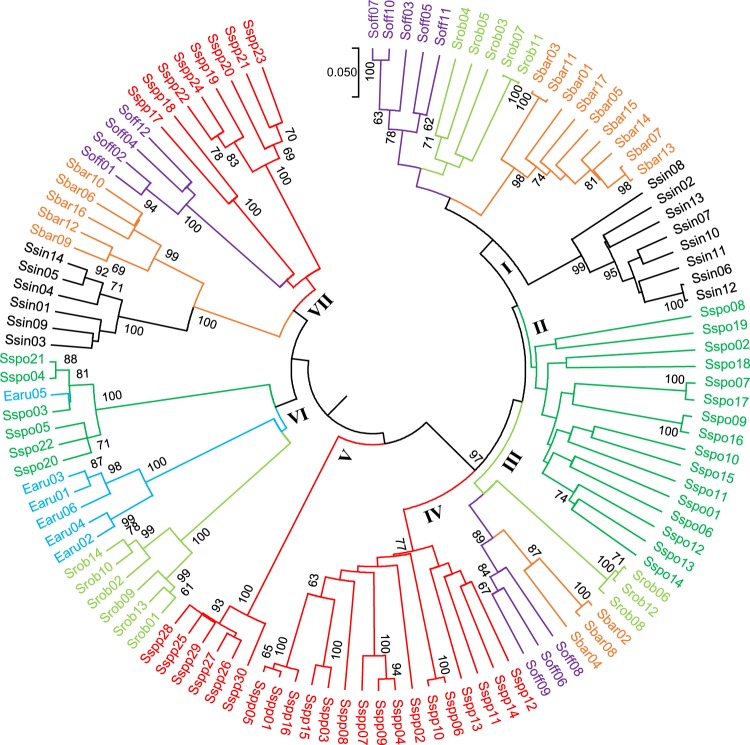
Phylogenetic trees of *Saccharum, Erianthus*, and *Saccharum* spp. hybrids based on SSR data. A distance tree was constructed in MEGA 6 using the UPGMA method. Robustness of the node of the phylogenetic tree was assessed from 1000 bootstrap replicates and bootstrap values of >60% are shown.

A phylogenetic tree is shown in Fig. 3. Based on phylogenetic analysis, the 115 accessions were clearly clustered at *Saccharum* and *Erianthus* genera level into seven major clades, also involving different *Saccharum* and *Erianthus* species to some extent. Clade-I contained 27 accessions from *S. officinarum, S. robustum, S. barberi* and *S. sinense*. Clade-II included 16 accessions from *S. spontaneum*. Clade-III comprised of three accessions of *S. officinarum*, three accessions of *S. robustum*, and three accessions of *S. barberi*. Clade-IV and Clade-V held 22 accessions of *Saccharum* spp. Hybrids. Clade-VI clustered 13 accessions of *S. robustum* and *S. spontaneum* and five accessions of *E. arundinaceus*. However, one *E. arundinaceus* accession, Guizhou 78-I-24 (Earu05), was clustered with six *S. spontaneum* accessions. Finally, Clade-VII contained eight accessions of *Saccharum* spp. hybrids, four accessions of *S. officinarum*, five accessions of *S. barberi*, and six accessions of *S. sinense*.

**Figure 4:**
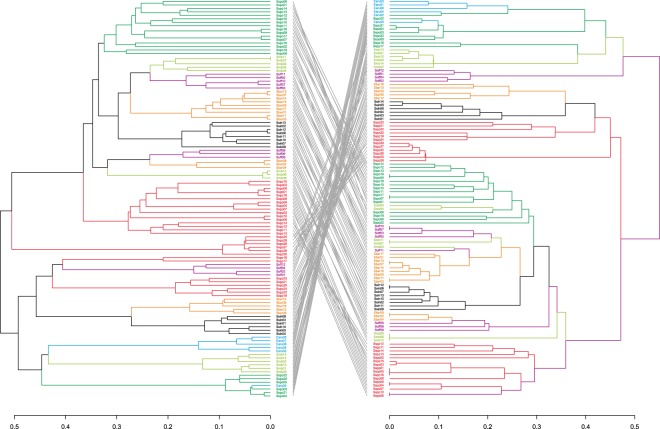
Two phylogenetic trees constrcuted using SSR data derived from 21 SSR primer pairs *vs* 7 SSR primer pairs with tanglegrams.

To verify some core SSR primer pairs out of the 21 primer pairs, we compared two phylogenetic trees constructed based on CE-data of 21 SSR primer pairs *vs* 7 SSR primer pairs and of 21 SSR primer pairs *vs* 6 SSR primer pairs with the Robinson-Foulds distance. Further analysis with Dendextend showed a higher cophenetic correlation coefficient value (0.93) between 21 SSR primer pairs and 7 SSR primer pairs than the 0.91 cophenetic correlation coefficient value between 21 SSR primer pairs and 6 SSR primer pairs. The plots of two phylogenetic trees based on the CE-data of 21 SSR primer pairs *vs* 7 SSR primer pairs are shown in Fig. 4 with tanglegrams.

### Genetic identity analysis

Percent of genetic identity was estimated between and within the seven phylogenetic groups. Percent genetic identity between phylogenetic groups ranged from 26.9% (*Saccharum* spp. hybrids and *S. spontaneum*) to 96.4% (*E. arundinaceus* and *S. spontaneum*). Percent genetic identity within phylogenetic groups ranged from 38.9% (within *S. barberi* or *Saccharum* spp. hybrids) to 100% (within *S. robustum*) (Table 3).

**Table 3:** Genetic identity (%) among five *Saccharum* species, one *Erianthus* species, and *Saccharum* spp. hybrids based on SSR data.

Populations	*S. spontaneum*	*S. officinarum*	*S. barberi*	*S. robustum*	*S. sinense*	*Saccharum* spp. hybrids	*E. arundinaceus*
*S. spontaneum*	98.8–39.5						
*S. officinarum*	74.8–34.7	97.6-39.5					
*S. barberi*	73.0–37.7	83.2–36.5	99.4–38.9				
*S. robustum*	79.0–36.5	83.2–37.1	79.0–37.1	100–431			
*S. sinense*	73.6–42.5	74.2–46.7	78.4–41.3	74.2–44.3	99.4–43.7		
*Saccharum* spp. hybrids	73.6–26.9	79.6–29.9	72.4–33.5	70.0–28.1	69.4–35.9	98.8–38.9	
*E. arundinaceus*	96.4–40.1	56.2–38.3	53.8–39.5	59.2–40.7	57.4–37.1	57.4–39.5	96.4–50.8

## Discussion

Since 1950s, wild accessions of *Saccharum* and *Erianthus* have been continuously collected on mainland China and maintained in the Sugarcane Germplasm Nurseries in Yacheng, Hainan province or Kaiyuan, Yunnan Province, China. However, the genetic relationship and molecular identification between these two germplasm collections have never been entirely examined. Molecular markers are considered to be most effective in analyzing the genetic diversity, population structure, and phylogenetic relationship within sugarcane germplasm^31^. In recent years, SSR markers are proven to be very useful for a variety of applications in plants, including linkage maps analysis, segregation analysis, population structure analysis, marker-assisted selection, assessment of genetic relationships between individuals, mapping genes of interest, and marker-assisted backcrosses, population genetics and phylogenetic studies^32,33^.

In this study, we investigated the genetic diversity and population structure for 115 accessions of *Saccharum* and *Erianthus* genera that originated from two collections on mainland China and a local collection in the USA by 21 SSR primer pairs. The 21 primer pairs primed the amplification of 167 polymorphic SSR alleles detectable by the CE platform, of which 38 alleles have never been reported before. Every primer pair was able to amplify varying numbers of SSR alleles from all accessions tested, regardless of their geographical origins. Seven core SSR primer pairs, namely, SMC24DUQ, SMC31CUQ, SMC336BS, SMC597CS, SMC703BS, mSSCIR3, and mSSCIR43, produced more than ten alleles among the 115 accessions, while four of the seven core primer pairs, namely, SMC31CUQ, SMC336BS, SMC597CS, and mSSCIR3, also primed the amplification of more than 10 alleles among 92 Chinese commercial sugarcane varieties^7^. Therefore, these seven core SSR primer pairs would have a priority of choice in identifying clones either from *Saccharum* species or *Saccharum* spp. hybrids.

The number of polymorphic SSR alleles detected in this study was higher than the 144 alleles reported by Pan^30^ or the 151 alleles reported by Ahmad *et al*.^7^, but lower than the 205 polymorphic alleles reported by You *et al*.^25^. We considered that the differences were due to different *Saccharum* clones being used in previous studies or to different scoring criteria. The differences may also be due to the complex genomes of *Saccharum* and *Erianthus* on one hand and relatively narrow genetic base of commercial sugarcane varieties on the other hand.

In this study, we observed different levels of genetic variations among accessions of *Saccharum* and *Erianthus* tested. In general, *Saccharum* spp. hybrids and *S. spontaneum* accessions had a higher genetic diversity than *S. sinense* and *E. arundinaceus* accessions. However, the highest number of observed alleles, number of effective alleles and polymorphism index were observed in accessions of *Saccharum* spp. hybrids, which are polyploidy with genome contributions from several *Saccharum* species. Historically, the modern *Saccharum* spp. hybrids were developed from crosses between the “Noble” cane *S. officinarum* and its relatives, namely, *S. spontaneum*, *S. sinense*, or *S. barberi* in the early 20th century^34,35^. The overall genetic variation values from this study were higher than those reported by You *et al*.^24,25^. We hypothesize that this phenomenon was due to the utilization of a larger number of SSR primer pairs and the large number of accessions from diverse *Saccharum* and *Erianthus* species in our study.

It is worthnoting that the 21 SSR primer pairs worked well in clustering *Saccharum*, *Erianthus*, and *Saccharum* spp. hybrids clones during phylogenetic analysis process. Two *Saccharum* spp. hybrids clones [(R570 (Sspp17)] from France and [(Q124 (Sspp18)] from Australia were clustered into a sub-clade in Clade-VII with four accessions of *S. officinarum*. The reason could be that R570 and Q124 varieties may have a closer affiliation with *S. officinarum*. In addition, the six accessions of *E. arundinaceus* were clustered with accessions of *S. robustum* and *S. spontaneum* in Clade-VI rather than forming a separate clade. This was because all the 21 SSR primer pairs were designed from the genomic DNA sequences of two cultivars, either Q124 or R-570^30^. Unlike some consensus primers that are able to prime the PCR amplification of plant genomic sequences^36^, these SSR primer pairs may not be able to amplify *Erianthus* genomic DNA at equivalent efficiency as they do to the *Saccharum* genomes. Another reason is that it is now generally accepted that Noble cultivars might directly emerge from *S. robustum*. It also has hypothesized that *S. robustum* be evolved from complex introgressions between *S. spontaneum* and other genera, particularly *Erianthus* and *Miscanthus* sharing close genetic affiliation^37,38^. The genetic diversity results from our study were in general conformity with the evolutionary course of the sugarcane cultivars in that the order of contributing species in today’s accessions is *S. officinarum*, *S. spontaneum*, *S. robustum*, *S. barberi*, *S. sinense*, *E. arundinaceus* and *Saccharum* spp. hybrids. PCA analysis also revealed a similar pattern of phylogeny to some extent.

Today, China holds more than 2,000 accessions of *Saccharum* and *Erianthus*, among which some are wild types. These accessions are either China-born or through foreign introductions. As the size of sugarcane germplasm grows, the genetic information among accessions becomes more critical for maintaining and utilization strategies designed to establish cross parentages in China’s breeding programs. We conclude that the estimation of genetic diversity and population structure of 115 accessions of *Saccharum* and *Erianthus* genera using SSR primer pairs may provide more accurate information to sugarcane breeders than the classical pedigree method. The 21 SSR primer pairs used in our study may also be of potential value for further research on genetic mapping, segregation analysis, marker-assisted selections, QTL mapping and gene tagging in sugarcane. In addition, further study with consensus PCR primers may be needed to assess the phylogenetic status of the *Erianthus* genus within the “*Saccharum* Complex”^38^.

## Materials and Methods

### Plant materials

One hundred and fifteen asseccions were used in this study, including 12 accessions from *S. officinarum*, 22 from *S. spontaneum*, 14 from *S. robustum*, 17 from *S. barberi*, 14 from *S. sinense*, 30 from *Saccharum* spp. hybrids, and six from *E. arundinaceus*. The leaf samples of all the clones were collected either from the Sugarcane Germplasm Nursery in Yacheng, Hainan, China or a local collection at the USDA-ARS, Sugarcane Research Unit, Houma, Louisiana, USA (Table 4). The leaf samples were collected, wiped off with 75% ethanol, and kept at −80 °C until DNA extraction.

**Table 4:** A list of 115 accessions from *Saccharum*, *Erianthus*, and *Saccharum* spp. hybrids.

No.	Accessions name	Sample no.	Species	No.	Accessions name	Sample no.	Species
1	48Mouna	Soff01	*S. officinarum*	59	Djatiroto	Sspo02	*S. spontaneum*
2	Badila	Soff02	*S. officinarum*	60	Fujian 79-I-1	Sspo03	*S. spontaneum*
3	Bandjermasin Hitam	Soff03	*S. officinarum*	61	Guangdong 29	Sspo04	*S. spontaneum*
4	Barwhspt	Soff04	*S. officinarum*	62	Guangdong 51	Sspo05	*S. spontaneum*
5	EK02	Soff05	*S. officinarum*	63	IMP9068	Sspo06	*S. spontaneum*
6	Fiji1	Soff06	*S. officinarum*	64	IMP9089	Sspo07	*S. spontaneum*
7	IN84-068B	Soff07	*S. officinarum*	65	IND81-080	Sspo08	*S. spontaneum*
8	LA Purple	Soff08	*S. officinarum*	66	Mol1032A	Sspo09	*S. spontaneum*
9	Muntok Java	Soff09	*S. officinarum*	67	Mpth97-107	Sspo10	*S. spontaneum*
10	NG21-003	Soff10	*S. officinarum*	68	Mpth97-233	Sspo11	*S. spontaneum*
11	NG57-223	Soff11	*S. officinarum*	69	PCANOR84-2A	Sspo12	*S. spontaneum*
12	Striped Cheribon	Soff12	*S. officinarum*	70	PCAV84-12A	Sspo13	*S. spontaneum*
13	51NG208	Srob01	*S. robustum*	71	PQ84-3	Sspo14	*S. spontaneum*
14	51NG63	Srob02	*S. robustum*	72	S66-084A	Sspo15	*S. spontaneum*
15	IJ76-339	Srob03	*S. robustum*	73	S66-121A	Sspo16	*S. spontaneum*
16	IN84-045	Srob04	*S. robustum*	74	SES323A	Sspo17	*S. spontaneum*
17	IN84-076	Srob05	*S. robustum*	75	SPONT24	Sspo18	*S. spontaneum*
18	M3035-66	Srob06	*S. robustum*	76	SPONT37	Sspo19	*S. spontaneum*
19	NG28-289	Srob07	*S. robustum*	77	Yacheng 11	Sspo20	*S. spontaneum*
20	NG57-055	Srob08	*S. robustum*	78	Yacheng 12	Sspo21	*S. spontaneum*
21	NG77-004	Srob09	*S. robustum*	79	Yunnan 82-114	Sspo22	*S. spontaneum*
22	NG77-1	Srob10	*S. robustum*	80	Fijian 87-II-5	Earu01	*E. arundinaceus*
23	NG77-159	Srob11	*S. robustum*	81	Guangxi 83-27	Earu02	*E. arundinaceus*
24	NG77-235	Srob12	*S. robustum*	82	Hainan 92-79	Earu03	*E. arundinaceus*
25	NG77-75	Srob13	*S. robustum*	83	Hainan 92-105	Earu04	*E. arundinaceus*
26	Teboe Salak Toewa	Srob14	*S. robustum*	84	Guizhou 78-I-24	Earu05	*E. arundinaceus*
27	Agoule	Sbar01	*S. barberi*	85	Sichuan 79-I-13	Earu06	*E. arundinaceus*
28	Chunnee	Sbar02	*S. barberi*	86	HoCP01-517	Sspp01	*S*. spp. hybrids
29	Dhaula	Sbar03	*S. barberi*	87	HoCP85-845	Sspp02	*S*. spp. hybrids
30	Hatuni	Sbar04	*S. barberi*	88	HoCP91-555	Sspp03	*S*. spp. hybrids
31	HulluKabbu	Sbar05	*S. barberi*	89	HoCP96-540	Sspp04	*S*. spp. hybrids
32	Kacai	Sbar06	*S. barberi*	90	L01-283	Sspp05	*S*. spp. hybrids
33	Keari	Sbar07	*S. barberi*	91	L01-299	Sspp06	*S*. spp. hybrids
34	Khagzi	Sbar08	*S. barberi*	92	L97-128	Sspp07	*S*. spp. hybrids
35	Mungo	Sbar09	*S. barberi*	93	L99-233	Sspp08	*S*. spp. hybrids
36	Nagans	Sbar10	*S. barberi*	94	LCP85-384	Sspp09	*S*. spp. hybrids
37	NEWRA	Sbar11	*S. barberi*	95	MEX85-6196	Sspp10	*S*. spp. hybrids
38	Pansahi	Sbar12	*S. barberi*	96	TCP93-4245	Sspp11	*S*. spp. hybrids
39	Pathri	Sbar13	*S. barberi*	97	TCP97-4442	Sspp12	*S*. spp. hybrids
40	Rounda	Sbar14	*S. barberi*	98	TCP98-4445	Sspp13	*S*. spp. hybrids
41	Ruckri	Sbar15	*S. barberi*	99	TCP98-4447	Sspp14	*S*. spp. hybrids
42	Sewari	Sbar16	*S. barberi*	100	US01-40	Sspp15	*S*. spp. hybrids
43	Sunnabile	Sbar17	*S. barberi*	101	US02-99	Sspp16	*S*. spp. hybrids
44	Binchuanxiaozhe	Ssin01	*S. sinense*	102	Q124	Sspp17	*S*. spp. hybrids
45	ChukChe	Ssin02	*S. sinense*	103	R570	Sspp18	*S*. spp. hybrids
46	Guangdongzhuzhe	Ssin03	*S. sinense*	104	ROC10	Sspp19	*S*. spp. hybrids
47	Guangxizhuzhe	Ssin04	*S. sinense*	105	ROC16	Sspp20	*S*. spp. hybrids
48	Merehi	Ssin05	*S. sinense*	106	ROC20	Sspp21	*S*. spp. hybrids
49	MiaLan	Ssin06	*S. sinense*	107	ROC22	Sspp22	*S*. spp. hybrids
50	Nepal3	Ssin07	*S. sinense*	108	ROC25	Sspp23	*S*. spp. hybrids
51	TanzhonBamboo	Ssin08	*S. sinense*	109	ROC27	Sspp24	*S*. spp. hybrids
52	Tanzhouzhuzhe	Ssin09	*S. sinense*	110	FN41	Sspp25	*S*. spp. hybrids
53	TekchaOkinawa	Ssin10	*S. sinense*	111	GT40	Sspp26	*S*. spp. hybrids
54	UbaDelNatal	Ssin11	*S. sinense*	112	MT01-77	Sspp27	*S*. spp. hybrids
55	UbaIndia	Ssin12	*S. sinense*	113	LC05-136	Sspp28	*S*. spp. hybrids
56	UbaNaquin	Ssin13	*S. sinense*	114	YT00-236	Sspp29	*S*. spp. hybrids
57	Wenshanzhuzhe	Ssin14	*S. sinense*	115	YZ05-51	Sspp30	*S*. spp. hybrids
58	Coimbatore	Sspo01	*S. spontaneum*				

### DNA extraction

Genomic DNA was extracted from leaf tissues using a modified cetyl tri-methyl ammonium bromide (CTAB) method^39^ as previously described by Ahmad *et al*.^7^. The quality and concentration of DNA were measured using UV absorbance assay with a Synergy™ H1 Multi-Mode Reader (BioTek, Winooski, VT, USA) and 0.8% agarose gel electrophoresis with ethidium bromide staining.

### SSR markers and SSR reactions

The 21 polymorphic SSR primer pairs from Pan^30^ were used in this study based on their high PIC values of greater than 0.78^7,40,41^. All forward primers were labeled with the fluorescence dye, 6-carboxy-fluorescein (FAM). Serials of PCR-cycling conditions were performed to detect the SSR DNA fingerprints^7,30^. The PCR products for the capillary electrophoresis (CE) were conducted on ABI 3730XL DNA Analyzer (Applied Biosystems Inc., Foster City, CA, USA) following the manufacturer’s instructions to generate GeneScan files.

### Marker scoring

The GeneScan files were analyzed with the GeneMarker™ software (version 1.80) (SoftGenetics LLC^®^, State College, PA, USA, www.softgenetics.com) to reveal capillary electrophoregrams of PCR amplified SSR-DNA fragments. Fragment sizes were computed automatically against the GS500 DNA size standards (Applied Biosystems, Inc., Foster City, CA, USA). SSR alleles were manually assigned to unique, true “Plus-adenine” DNA fingerprints that gave quantifiable fluorescence values. Irregular peaks and stutters peaks were not scored according to Pan *et al*.^41^. Data were scored manually in a binary format into a data matrix file, with the presence of a band scored as “1” or “A” and its absence scored as “0” or “C”. The polymorphism information content (PIC) values were calculated using the formula of Liu *et al*.^23^.

$$PIC=1-\sum _{J=1}^{n}\,{P}_{ij}^{2}$$

Where *P*_*ij*_ is the frequency of *j*_th_ allele for *i*_th_ locus and summation extends over *n* alleles.

### Data analysis

The allelic data matrix of “1” or “0” was used to calculate the population genetic analysis using POPGENE version 1.32^42^, including number of observed alleles (*Na*), and number of effective alleles (*Ne*). Nei’s genetic diversity (*h*), polymorphism index (*PI*) and Shannon’s index (*I*) were computed for each *Saccharum* and *Erianthus* populations based on the obtained allele frequencies. The allelic data matrix of “A” or “C” was used to perform phylogenetic analysis. Phylogenetic tree was constructed with MEGA 6 using UPGMA statistical method with substitution model of Maximum Composite Likelihood^43^. Robustness of the node of the phylogenetic tree was assessed from 1000 bootstrap replicates. To find out the core-primer pairs of 21 SSR primer pairs, two other phylogenetic trees were constructed using SSR data from six or seven highly polymorphic SSR primer pairs. Then, the three phylogenetic tree files were calculated and Robinson-Foulds distances of 21 SSR *vs* 7 SSR and 21 SSR *vs* 6 SSR determined with Phangorn Package^44^ and cophenetic correlation coefficients of the topological distance were analyzed with Dendextend^45^. To better view the comparison between trees, Dendextend were used to plot two trees with tanglegrams. Genetic identity matrix was calculated using BioEdit Sequence Alignment Editor Version 7.1.9^46^. Genetic similarity coefficients among *Saccharum* and *Erianthus* populations were estimated with the SIMQUAL subprogram using the Jaccard’s coefficient, followed by principal component analysis (PCA) with the DICE subprogram as implemented in NTSYS-pc version 2.10e^47^.
